# Are There Adverse Events after the Use of Sexual Enhancement Nutrition Supplements? A Nationwide Online Survey from Japan

**DOI:** 10.3390/nu11112814

**Published:** 2019-11-18

**Authors:** Chiharu Nishijima, Etsuko Kobayashi, Yoko Sato, Tsuyoshi Chiba

**Affiliations:** Department of Food Function and Labeling, National Institute of Health and Nutrition, National Institutes of Biomedical Innovation, Health and Nutrition, 1-23-1 Toyama, Shinjuku-ku, Tokyo 162-8636, Japan; c-nishijima@nibiohn.go.jp (C.N.); e-kbyshi@nibiohn.go.jp (E.K.); satoyoko@nibiohn.go.jp (Y.S.)

**Keywords:** dietary supplement, sexual enhancement, adverse events, internet survey

## Abstract

Dozens of safety alerts for sexual enhancement and weight loss dietary supplements have been launched from the government not only in Japan but also overseas. However, adverse events have been reported only for the use of weight loss supplements, and the prevalence of use and adverse events in sexual enhancement supplements is not known in Japan. To address this issue, we assessed the situation of sexual enhancement supplement use through a nationwide online survey. The prevalence of sexual enhancement supplement use among males was 23.0%. Use of these supplements was higher among younger people than among older people (*p* < 0.001). In total, 17.6% of users had experienced adverse events, but 58.3% of them did not consult about the events with anybody because of the temporality of their symptoms and their sense of shame. In addition, eight supplement products were found to be possible adulterated supplements in this survey. It is necessary to inform the public about the risk of sexual enhancement supplement use and also prepare a place for consultation on media channels that younger people are more familiar with, in order to monitor adverse events while also preserving their privacy.

## 1. Introduction

The use of dietary supplements has expanded worldwide. With the increase in the health consciousness of the public, numerous dietary supplements claiming to support physical function have been developed and sold in Japan. Consumers expect to gain health benefits from these supplements, but there are inherent risks of adverse effects caused by the ingredients themselves and interactions with medicinal drugs [1]. In addition, herbal products that illegally have undeclared synthetic substances or adulterants added to them to gain increased impact and fast action have been found on the market [2]. Health and food safety related authorities in each country, such as the U.S. Food and Drug Administration (FDA), the European Food Safety Authority (EFSA), and the Ministry of Health, Labour and Welfare in Japan, take measures to investigate these adulterated products and take actions to remove them from the market. At the same time, they also issue public notices and safety alerts to protect consumers from these products.

In Japan, although adulterated supplement products are mainly found overseas, the current media channels provide us with easy access to purchase these products. Therefore, we have been working to disseminate public notices and safety alerts issued around the world through the online database, “Information system on safety and effectiveness for health foods (Japanese only)” [3,4]. Previously, we reported that 85% of safety alerts launched from the USA, EU, and Asia Pacific from 2010 to 2016 consisted of recall or warning information about food products containing undeclared medicinal ingredients [5]. Of these food products, sexual enhancement supplement products accounted for the highest proportion (36.8%) followed by weight loss supplements (31.3%). A similar situation has been seen in the USA, with 45.5% of adulterated dietary supplements being marketed for sexual enhancement, according to a warning released by the FDA, from 2007 to 2016 [6].

The most common adulterants found in sexual enhancement supplements are phosphodiesterase type five inhibitors (PDE5i), known as sildenafil, vardenafil, tadalafil, and their analogs [2,6]. The adverse effects of PDE5i are mostly mild with the occurrence reported to be 37.31% compared with 24.03% for placebo, but serious and fatal symptoms have also been reported in 1.05% to 1.85% of men treated with PDE5i [7,8]. In addition, the drug-drug interactions between PDE5i and nitrates are well known to lower blood pressure to dangerous levels [6]. Moreover, some of the adulterated sexual enhancement supplements contain more than one active pharmaceutical ingredient [2,6]. Some botanical ingredients used for sexual enhancement supplements were also reported to be associated with adverse events [9,10,11,12]. Thus, there is a tremendous possibility of adverse events when considering drug-botanical interactions. However, a majority of alerts on adverse events that have occurred in Japan were associated with the use of adulterated weight loss supplements by young females, and no adverse event cases associated with sexual enhancement products were included [5].

Meanwhile, Japanese government collects information on adverse events related to dietary supplements mainly from consumers and physicians through spontaneous reporting systems, but only about 20 severe cases are collected per year [13]. This underreporting is attributed by both consumers and health professionals; (1) consumers do not report the incidents anywhere or seek for clinical care because the symptom is not severe and they also do not want to tell physicians about the supplement use and (2) physicians and pharmacists do not report the incidents because it is difficult to define causal relationship or because the symptom is minor [14]. Thus, we previously conducted nationwide surveys on consumers directly to collect incidences of minor health damages, and that showed approximately 4% and 0.8% of supplement users experienced diarrhea and skin manifestation, respectively [15,16,17]. Another report showed that 8.6% of patients who used supplements concomitantly with medicines experienced health damages such as diarrhea, headache, rash, and effects on health examination data [18]. In addition to dietary supplements, it was reported that the occurrence of adverse events caused by weight management drugs was 5.4% (abdominal pain) to 39.3% (nausea) in the case of Lorcaserin [19]. This ratio was almost two to three times compared to placebo.

Since the use of sexual enhancement supplements is not well researched, the reason why adverse events are not notified is not known; therefore, to investigate whether adverse events related to the use of sexual enhancement supplements have occurred in Japan, and if they do occur, why there have been no cases publicly notified, a nationwide online survey was conducted. In this study, we hypothesized two possibilities: (1) the prevalence of the use of sexual enhancement supplements is low and (2) a sense of shame inhibits people from consulting about or reporting adverse events. Since cases related to weight loss supplements have been occasionally alerted about, unlike ones related to sexual enhancement supplements, females who used weight loss supplements were also surveyed as a counterpart to males who used sexual enhancement supplements.

## 2. Materials and Methods

### 2.1. Online Survey and Study Population

The present study was approved by the Research Ethics Committee of the National Institutes of Biomedical Innovation, Health and Nutrition (No. 28; approval date: 31 January 2019) and was conducted in accordance with the Declaration of Helsinki. An internet survey was administered from 12 to 22 February 2019 by a survey research company, Cross Marketing Inc. (Tokyo, Japan).

The research consisted of a preliminary survey to screen targeted participants who use sexual enhancement supplements in males and weight loss supplements in females. In the preliminary survey, we added a trap question to eliminate the fraudulent and professional respondents who skip reading questions [20]. The trap question was “Have you used a supplement for revitalization purpose?” and directed the participants to select three (I used it for this purpose one to three years before) regardless of their actual situation. The participants of the preliminary survey who correctly answered the trap question were eligible to go on to the actual survey, where details about supplement use were asked. The research company sent an invitation e-mail to its registrants over 20 years old to participate in the survey and collected responses on a first come, first served basis, until the number reached our request of approximately 1000 responses from the targeted population of each sex. The quota limits based on Japanese population were not applied. In total, 31,791 males and 29,316 females responded to the preliminary survey. Of these respondents, supplement users were 18,040 males and 19,928 females. The users of supplements for sexual enhancement and weight loss were 4147 males and 9792 females, respectively. Among them, those who passed the trap question were invited to the actual survey. Finally, 926 males and 987 females completed all the questions.

### 2.2. Questionnaires

Throughout the survey, a dietary supplement was defined as a food product in tablet, capsule, or liquid form that was sold and used to gain beneficial effects for human health. We also stated that medical drugs and products in the form of conventional foods (e.g., beverages and yogurt) that claimed a specific function were not included.

In the preliminary survey, participants were asked about their experience of dietary supplement use. Respondents who answered that they were “currently using” or had “previously used” supplements were considered to be “supplement users” and proceeded to the next five questions plus a trap question, which asked about their supplement use for specific purposes. Five purposes were selected from the common responses in our previous studies [18,21]: “supplementation of nutrients”, “muscle enhancement”, “disease prevention or treatment”, “revitalization” (trap), and “no special reasons”, along with “sexual enhancement” for males and “weight loss” for females. Those who currently or previously used sexual enhancement or weight loss supplements proceeded to the actual survey.

In the actual survey, the following questions were asked regarding the use of sexual enhancement (males) or weight loss (females) supplements: Usage of supplements (frequency, medical status, and sources of information), the name of the product and manufacturer (required to type in at least one and up to three names) and the channels of acquisition of each product, adverse event experience, and the attitude toward consulting and reporting about adverse events. For those who did not experience any adverse events, we also asked about the attitude toward consulting and reporting about adverse events by assuming if they were to experience adverse events such as palpitation or shortness of breath. For those who answered that they did “not consult with anybody about adverse events”, they were also asked about their reason. Lastly, all the participants were asked who they would not want to know about their use of sexual enhancement or weight loss supplements.

### 2.3. Analysis of the Survey Data

The supplement products that the participants used were analyzed by categorizing them into types (supplements, drugs, and products otherwise specified) and classifying them into major ingredients. The answers of “don’t know” or “don’t remember” the name of the product and manufacturer were excluded from the analysis (*n* = 144 in males and *n* = 99 in females). Advertising claims and ingredient information were obtained from the websites of the manufacturer or retail stores by Google searching the name of the product and manufacturer.

The major ingredients of sexual enhancement products were animal and botanical derived preparations; amino acids; energy products, which contained caffeine, vitamins, and minerals; and others, which included small numbers of resveratrol, royal jelly, sesamin, and coenzyme Q10. For weight loss products, the major ingredients were animal and botanical derived preparations; amino acids/proteins; exercise-supporting combination products, which included creatine and *β*-hydroxy-*β*-methylbutyrate, vitamins, and minerals; and others, which included small numbers of white kidney bean, coenzyme Q10, capsaicin, and Garcinia. Botanicals and animals were further classified by their major single ingredient, and the rest were named as combination products.

Categorical valuables were aggregated and expressed as percentages. Since up to three products were reported per participant, the cumulative number was counted after classification into types and major ingredients and expressed as a number and a percentage. Differences among groups, such as age and gender, were examined using the Chi-square test. Statistical analyses were performed using JMP (SAS Institute Inc., Cary, NC, USA) version 13.0 with the statistical significance set to *p* < 0.05.

## 3. Results

### 3.1. Prevalence of Dietary Supplement Use

The prevalence of dietary supplement use in the preliminary survey overall was 56.7% (33.6% current users and 23.1% previous users) in males and 68.0% (38.5% current users and 29.5% previous users) in females. Table 1 shows the prevalence according to age. The proportion of current users increased along with age, and this trend was more evident in females. The current and previous supplement users (males, *n* = 18,040; females, *n* = 19,928) were further asked about their use of supplements for specific purposes. The prevalence of users of sexual enhancement supplements in males and weight loss supplements in females is shown in Table 2. In males, 9.1% of male supplement users were currently using sexual enhancement supplements, and 13.9% of them had used them previously overall, but the proportion of both current and previous users decreased along with age; thus, there were more users in younger age groups. In females, the prevalence of current users of weight loss supplements was stable at around 13.5% among the ages of 20s to 50s. Approximately half of female supplement users used them for weight loss purposes currently or previously.

### 3.2. Participants in the Actual Survey

In the actual survey, 926 males who currently or previously used sexual enhancement supplements and 987 females who currently or previously used weight loss supplements answered the questionnaire. The frequency of use was less than one–two days/week in half of males, whereas most females took supplements almost every day. Table 3 shows the physical status related to the use of sexual enhancement/weight loss supplements. In males, more people in younger age groups visited a clinic (*p* = 0.001) and were on medication to improve sexual function (*p* = 0.018). More people in older age groups were on medication for other purposes (*p* = 0.007). In females, more younger people tried to improve life habits while using a weight loss supplement (*p* = 0.023). A small percentage of people, but more people, in younger age groups were on medication to lose weight (*p* = 0.014). Older age groups were more likely to be undergoing treatment for other diseases (*p* < 0.001).

### 3.3. Sources of Information About Sexual Enhancement/Weight Loss Supplements

In males, over half gained information from the internet in all age groups, but younger males relied more on individuals’ experiences and reviews on the internet and on social media than older age groups (*p* = 0.017 and *p* < 0.001, respectively) (Table 4). On the other hand, males in their 60 s used newspapers, magazines, and flyers as an information source (*p* < 0.001). In females, information on the internet was referred to the most in all age groups, but females in their 60 s gained information from television and radio the most (*p* < 0.001). Newspapers, magazines, and flyers were used among females in their 60 s, more so than younger age groups, who used reviews on social media (*p* = 0.014 and *p* < 0.001, respectively); the proportions of users of the internet and users of reviews on social media were equal (45.3%) among females in their 20 s.

### 3.4. Types and Major Ingredients of Sexual Enhancement/Weight Loss Supplement Products

We asked participants to report up to three products; thus, the cumulative number of sexual enhancement products was 1062 (Table 5). Of all the products, supplements comprised 68.1%, and 12.7% were defined as drugs. Among supplement products, the most used ingredients were animal and botanical (536/847, 63.3%) followed by amino acids (12.0%), vitamins (4.8%), zinc, and other minerals (4.3%). Amino acids consisted almost entirely of arginine, citrulline, and ornithine. Among animal and botanical ingredients, Maca (15.9%) was the most popular single ingredient and was also used in most of the combination products. The combination products (68.8%) were mixtures of the listed single ingredients and other botanicals, such as guarana, Tongkat Ali, red Kwao Keur, and saw palmetto, and animals, such as Chinese softshell turtle, seal bear, freshwater clam, and Japanese pit viper, and amino acids, vitamins, and minerals. Of all the products, eight (0.8%) matched the names of adulterated supplements that had been alerted about as illegal products in Japan before.

The cumulative number of weight loss products was 1224; supplements and drugs comprised 88.1% and 3.3%, respectively (Table 6). The major ingredients of supplements were mostly animals and botanicals (83.9% of supplements) followed by amino acids/proteins (7.4%). Among animal and botanical ingredients, *Coleus Forskohlii* was the most used (16.0%) single ingredient followed by fermented botanical extracts (12.4%). Combination products, which contained animal and botanical preparations such as *Gymnema sylvestre*, white kidney bean, *Coleus Forskohlii*, chitosan, and *Salacia* species, consisted of 49.1% animals and botanicals. There was no product name matched to the illegal products that had been alerted about in Japan, but one respondent reported the use of a foreign product that contained Senna leaves, which are categorized as a drug ingredient and prohibited for use as a supplement product in Japan.

### 3.5. Acquisition of Products

Table 7 shows the channels of acquisition of each product according to age groups. In both males and females, the major channels of acquisition were stores and domestic online shopping; however, there was a trend that younger age groups were more likely to purchase sexual enhancement/weight loss supplements at stores, whereas older age groups were more likely to purchase them through domestic online shopping. The “Others” category in males was predominantly comprised of “at a clinic”, whereas in females the sources were various but esthetic beauty salons were the most reported. “Purchased when traveled overseas” and “received as a souvenir from overseas” were also reported by several participants among both males and females.

### 3.6. Adverse Event Experience

The prevalence of adverse event experience was 17.6% in males and 12.3% in females (Table 8). In detail, drug users experienced adverse events (males, 38.6%; females, 22.5%) more than supplement users; thus, the frequency of adverse events only due to the use of supplements for sexual enhancement was 11.2%, and for weight loss was 8.8%. Compared to females, warmth or redness in the face, neck, or chest were 10 times more reported and headaches were two times more reported in males (*p* < 0.001 and *p* = 0.010, respectively). In males, a relatively small number, but still 9.2% to 9.8%, reported palpitation or shortness of breath, fatigue, and dizziness. In females, gastrointestinal symptoms accounted for 53.7% of adverse symptoms, followed by nausea or heartburn (25.6%), and cutaneous symptom (11.6%).

### 3.7. Attitudes toward Consulting and Reporting about Adverse Events

Actions in response to adverse events (participants have done/will do), such as attitudes toward consulting and reporting, are shown in Table 9. “Not consult with anybody” was the most common answer in both males and females who had experienced adverse events (58.3% and 65.3%, respectively). Regarding the reasons why they would not consult with anybody, “the symptom is temporal” was the most reported in both males (51.6%) and females (53.2%). Meanwhile, more males selected “it is embarrassing” than females (44.2% and 12.7%, respectively; *p* < 0.001). Other reasons included “the symptoms became better by stopping the use”, “I already knew about the adverse symptom from the reviews on the Internet”, and “I was not sure that the adverse event was because of the product”. On the other hand, the retail store (males, 12.9%; females, 9.1%), family members, friends, relatives or colleagues (12.9%; 10.7%), and governmental agencies (9.8%; 3.3%) were the places where participants reported the incident after adverse events. A quarter of males and 30.7% of females who had never experienced an adverse event thought that they would consult with a doctor (clinic), but only 1.2% of males and 3.3% of females consulted with a doctor (clinic) among those who had experienced adverse events. Similarly, 20.1% of males and 35.2% of females who had never experienced an adverse event reported that they would ask or complain to the manufacturer, but much less people asked or complained to the manufacturer after an actual adverse event (males, 8.0%; females, 5.8%).

Among the participants with adverse event experience, approximately half of females had nobody who did not want to know about their use of weight loss supplements, while the corresponding proportion in males was only 21.5% (*p* < 0.001) (Table 10). Males did not want their family members or relatives (45.4%) and partner (37.4%) to know about their use of sexual enhancement supplements. Although it was a small proportion, there were males who did not want doctors and someone at the consultation services to know about their use (9.8% and 5.5%, respectively).

## 4. Discussion

In this study, we explored why there have been no safety alerts about adverse events related to sexual enhancement supplements among males, while alerts about weight loss supplements among females have been occasionally launched. Our data showed that the prevalence of sexual enhancement supplement use in males was less than half of the weight loss supplement use in females, but 23% of male supplement users had used supplements for sexual enhancement currently or previously. Although adverse events were experienced by 17.6% of sexual enhancement users, half of them were not consulted or reported to anybody due to the temporality of the symptoms and their sense of shame.

Dietary supplements for sexual enhancement were most used among males aged 20–29 years, with a decreasing trend according to age advancement, even though the prevalence of erectile dysfunction (ED) increasing in correlation to age has been previously reported [22,23,24]. The proportion of moderate-to-severe ED among men who lived with their spouses varied from 1.8% in the age group of 20–29 years to 64.3% in those over 70 years [22]. In accordance with the increasing trend of ED, the use of PDE5i to treat ED increased with age, especially among sexually active older-aged men [23]. There are several speculations about the highest use of sexual enhancement supplements observed in males in their 20s in our study. First, young men use these supplements for reasons other than ED treatment. An increasing demand for PDE5i by young men without ED to enhance sexual performance for recreational purposes in recent years has been observed [25], and higher awareness of ED was one of the independent predictors of seeing healthcare providers [26]. Therefore, younger males may casually try sexual enhancement supplements for enjoyment. Second, younger males were more likely to visit a clinic when they used supplements compared to older men, because there was a high possibility that they had not been diagnosed with ED and were not prescribed PDE5i drugs. In this case, they might try to use these supplements. Third, sexual enhancement supplements are sold with attractive sales copies. Younger males have more opportunities to find these sales copies as well as individuals’ experiences and reviews on websites and social media compared to older men; thus, they may be more tempted to try talked-about products.

The prevalence of adverse events due to sexual enhancement supplements was not as frequent as that among drug users, but similar symptoms were reported. Although adverse effects of supplement ingredients are often not fully understood, people often believe in the safety of these ingredients because they are just foods [18]. However, a majority of sexual enhancement supplements contained multiple concentrated food ingredients, and not only is the interaction between those ingredients not assured, but also some of them are associated with serious adverse events by themselves; for example, saw palmetto was associated with the development of thromboembolic events and allergic reactions [9], ginseng induced anaphylaxis [10], chromium caused acute tubular necrosis [11], and freshwater clam was related to the development of acute cholestasis [12]. If users experience adverse events from prescribed medicines, they have opportunities to consult with doctors or pharmacists. On the other hand, there are few opportunities to consult with physicians in the case of the use of dietary supplements.

Our results showed that supplement users for both sexual enhancement and weight loss thought that they would consult with a doctor if adverse events occurred. However, most of the users who had experienced adverse events did not consult with anybody. The adverse symptoms most commonly experienced due to the use of weight loss supplements by females were gastrointestinal symptoms, which we suppose the users could handle by themselves [15]. Since diarrhea accounts for the most cases among gastrointestinal symptoms related to weight loss supplements in general, some of the users may have thought that they could lose weight by enduring the diarrhea symptom. However, the symptoms experienced due to the use of sexual enhancement products were mainly flushing and included similar symptoms to those reported for PDE5i, including palpitation and dizziness. This was partly because drug users who experienced adverse events were included in the analysis, yet these users also did not consult about the symptom with anybody because the symptom was temporal and they felt embarrassed. Since they participated in this survey to answer about “dietary supplements”, they may have obtained the products without prescription. Furthermore, purchasing PDE5i without prior healthcare professional consultation was associated with embarrassment about speaking to a physician [27]. Therefore, consulting with a doctor about the adverse events related to the use of sexual enhancement products could also be embarrassing for them. It is necessary to prepare a place for consultation without direct contact.

The public notices issued in Japan about sexual enhancement products were mostly for imported products from overseas [5]. In our study, younger males in particular had direct access to foreign products through online shopping and friends in overseas. The issues related to the use of imported products are the inability to distinguish adulterated products and inability to return products when recalled—some ingredients are illegal for use as supplements in Japan, although they are legal in the manufactured country, such as the Senna leaves found in this study. Younger males were the ones who engaged the most in the use of sexual enhancement supplements. In addition, younger females were the ones most often affected by serious adverse events caused by imported adulterated weight loss supplements. Therefore, cautions and public notices should be disseminated through media channels that younger people use as information sources.

One of the findings in this study was that there were approximately 10% of users who used PDE5i drugs, although we defined “dietary supplements” and clearly stated that “drugs are not included” prior to the survey. Consumers often confuse supplements with drugs due to their similar forms; thus, they may not have perceived a difference between supplements and drugs. PDE5i drugs are known to be metabolized mainly by CYP3A4, and drug-drug interactions with potent CYP3A4 inhibitors and inducers have been a cause for concern [7,28,29]. Since a high proportion of ED patients have comorbidities such as hypertension and diabetes [23], there should be a warning for concomitant use of certain drugs with PDE5i [6]. Moreover, the concerns about interaction with PDE5i can be expanded to the concomitant use of dietary supplements containing food ingredients in high concentrations, because some ingredients, such as ginseng [30], garlic [31], and ginkgo [32], used for sexual enhancement were reported to influence the kinetics of drugs that are metabolized by CYP3A4.

There are several limitations related to the nature of the survey method. Since participants were registrants of the survey company who were particularly familiar with current communication technology, the participants may not represent Japanese people in general; the results may be biased to Internet users. Since supplements for sexual enhancement are sold via the Internet, the prevalence of supplement users might be larger in this study than that of general population. In addition, the participants were self-selected and willing to participate this survey among registrants of the survey company. The participants of the actual survey were those who passed the trap question. Indeed, approximately 78% of males and 90% of females were eliminated by this trap; thus, the trap question may have created more biases [20]. The results of this study may not reflect general population. Also, quota limits were not applied based on the population when collecting survey participants, and this may have influenced the data because regional differences were reported regarding the source of obtainment of PDE5i [26]. The data presented here were all self-reported; thus, symptoms of adverse events and causal relations to supplements were not medically examined, and the involvement of other foods, drugs, or diseases is unclear. Besides, the detailed situation of dietary supplement use was not surveyed. The adverse events experienced may be due to misuse of supplements such as over-use and concomitant use with other supplements. Moreover, since sexual function status was not asked about in this survey, the intensity of needs and the usage of supplements cannot be determined. The questionnaire also needs to be reexamined to make it better to collect information more effectively in the further study. However, despite these limitations, the prevalence of sexual enhancement supplement use and attitudes toward adverse events in this study is considerable information. Our findings suggest the need for a place for consultation and dissemination of warnings on media channels that younger people are more familiar with, in order to monitor adverse events related to sexual enhancement supplements.

## 5. Conclusions

Adverse event experiences among male users of sexual enhancement supplements were observed, contrary to the current situation where there have been no public notices regarding adverse injuries related to these supplement products. Supplement use was observed particularly in younger age groups, who obtained information from the Internet and social media. Since a majority of them did not consult about the adverse events with anybody, including physicians, due to embarrassment, a new approach to notify about the risks of these supplements should be applied, and adverse events should be monitored.

## Figures and Tables

**Table 1:** Prevalence of dietary supplement use according to age group in the preliminary survey.

	20–29	30–39	40–49	50–59	60–69	*p*-Value
Males, number	4668	6868	7342	6583	6330	
Current users (%)	26.5	32.7	32.8	35.5	38.9	<0.001
Previous users (%)	22.0	23.4	24.3	23.7	21.6	
Non-users (%)	51.6	43.8	42.9	40.8	39.5	
Females, number	7328	6346	5569	5778	4295	
Current users (%)	28.1	36.6	39.3	46.0	47.6	<0.001
Previous users (%)	30.5	33.9	30.8	26.6	23.6	
Non-users (%)	41.3	29.5	29.8	27.4	28.8	

Note: Total number of males was 31,791 and females was 29,316, expressed as percentage. The differences among age groups were examined by Chi-square test (*p* < 0.05).

**Table 2:** Prevalence of use of sexual enhancement supplements in males and weight loss supplements in females among supplement users.

	20–29	30–39	40–49	50–59	60–69	*p*-Value
Sexual enhancement in males, number	2261	3857	4192	3899	3831	
Current users (%)	10.0	10.9	8.8	8.6	7.4	<0.001
Previous users (%)	18.9	16.3	13.4	12.1	11.1	
Never used (%)	71.1	72.8	77.8	79.3	81.5	
Weight loss in females, number	4298	4472	3908	4194	305	
Current users (%)	12.8	13.8	14.3	13.2	8.0	<0.001
Previous users (%)	41.0	42.4	38.3	33.4	23.4	
Never used (%)	46.3	43.8	47.3	53.5	68.7	

Note: Total number of supplement users (current and previous) in males was 18,040 and in females was 19,928, expressed as percentage. The differences among age groups were examined by Chi-square test (*p* < 0.05).

**Table 3:** Physical status related to the use of sexual enhancement (males)/weight loss (females) supplements according to age groups.

	Sexual Enhancement (Males)						Weight Loss (Females)					
	20–29	30–39	40–49	50–59	60–69	*p*-Value	20–29	30–39	40–49	50–59	60–69	*p*-Value
number	130	239	198	175	184		225	259	199	214	90	
Trying to improve life habits (%)	46.9	38.9	46.0	29.1	38.0	0.006	64.9	60.2	54.3	53.7	47.8	0.023
Visited a clinic to improve sexual function/lose weight (%)	16.9	13.8	12.6	8.6	3.8	0.001	3.1	2.7	3.0	0.9	1.1	0.464
On medication to improve sexual function/lose weight (%)	20.0	19.7	15.7	10.9	10.3	0.018	8.4	4.2	3.0	3.3	1.1	0.014
Undergoing treatment for other diseases (%)	5.4	9.2	5.1	6.3	9.2	0.310	3.1	5.0	4.5	11.7	15.6	<0.001
On medication for other purposes (%)	6.2	5.4	8.6	12.6	14.7	0.007	7.1	8.5	11.1	13.1	14.4	0.135
None of the above (%)	28.5	36.0	30.3	48.0	37.5	0.002	22.2	30.1	36.2	33.2	36.7	0.014

Note: Multiple answers, expressed as percentage. The differences among age groups were examined by Chi-square test (*p* < 0.05).

**Table 4:** Sources of information about sexual enhancement (males)/weight loss (females) supplements according to age groups.

	Sexual Enhancement (Males)						Weight Loss (Females)					
	20–29	30–39	40–49	50–59	60–69	*p*-Value	20–29	30–39	40–49	50–59	60–69	*p*-Value
number	130	239	198	175	184		225	259	199	214	90	
Television, radio (%)	17.7	17.6	15.2	17.7	21.7	0.577	28.0	37.5	36.2	49.1	55.6	<0.001
Newspapers, magazines, flyers (%)	10.0	15.5	14.6	14.3	27.2	<0.001	13.3	17.4	22.1	22.9	27.8	0.014
Information on the internet (%)	50.0	51.0	58.6	60.6	59.2	0.132	45.3	51.4	52.3	51.4	47.8	0.576
Individuals’ experiences/reviews on the internet (%)	28.5	21.3	20.7	18.3	13.0	0.017	22.2	21.2	23.6	17.3	13.3	0.208
Reviews on social media (%)	21.5	20.5	13.6	6.9	5.4	<0.001	45.3	25.5	14.6	12.6	8.9	<0.001
Stores (point-of-purchase displays) (%)	10.8	19.2	12.1	14.9	10.9	0.068	20.4	27.8	22.6	30.4	25.6	0.121
Clinics (physicians, pharmacists, dietitians) (%)	9.2	9.6	7.6	4.6	3.3	0.055	2.2	3.1	1.5	0.9	0.0	0.263
Pharmacists or drugstore clerks (%)	11.5	14.2	9.1	5.7	7.6	0.038	7.6	8.9	8.5	7.9	5.6	0.887
Product labels (%)	6.9	15.5	11.6	6.3	7.1	0.006	18.2	20.8	23.1	21.5	13.3	0.340
Family, friends or acquaintances (%)	10.0	10.9	5.1	5.1	6.5	0.078	14.2	13.1	12.6	10.3	12.2	0.797
Others (%)	0.8	0.8	1.5	1.7	1.1	0.901	1.8	3.1	3.0	4.7	3.3	0.550

Note: Multiple answers, expressed as percentage. The differences among age groups were examined by Chi-square test (*p* < 0.05).

**Table 5:** Types and major ingredients of sexual enhancement products (males).

	Cumulative Number	% of All Products	% Among Animals and Botanicals
Supplements	847	68.1	
Animals and botanicals	536	43.1	
Maca	85		15.9
Garlic	37		6.9
French marine pine bark	19		3.5
Ginseng	12		2.2
Turmeric and ginger	7		1.3
Oyster	7		1.3
Combination products	369		68.8
Amino acids	102	8.2	
Vitamins	41	3.3	
Zinc and other minerals	36	2.9	
Energy products	28	2.3	
Omega-3	16	1.3	
Others	88	7.1	
Illegal drug products	8	0.8	
Products not otherwise specified	57	4.6	
Drugs	158	12.7	

Note: The number of male participants was 926. Of these participants, 144 were excluded for not remembering the product and manufacturer name. The total cumulative number of products was 1062; 591 participants reported one product, 102 reported two, and 89 reported three.

**Table 6:** Types and major ingredients of weight loss products (females).

	Cumulative Number	% of All Products	% Among Animals and Botanicals
Supplements	1078	88.1	
Animals and botanicals	904	73.9	
*Coleus Forskohlii*	145		16.0
Fermented botanical extracts ^1^	112		12.4
*Salacia* species	54		6.0
Probiotics	34		3.8
Vinegar products ^2^	26		2.9
Aojiru products ^3^	21		2.3
Chitosan	16		1.8
*Gymnema sylvestre*	15		1.7
Kudzu flower	12		1.3
Resistant starch	10		1.1
Tea ^4^	8		0.9
Sweet clover	7		0.8
Combination products	444		49.1
Amino acid/protein	80	6.5	
Vitamins and minerals	25	2.0	
Exercise-supporting combination products	9	0.7	
Others	60	4.9	
Products not otherwise specified	106	8.7	
Drugs	40	3.3	

Note: The number of female participants was 987. Of these participants, 99 were excluded for not remembering the product and manufacturer name. The total cumulative number of products was 1224; 661 participants reported one product, 118 reported two, and 109 reported three. ^1^ Aqueous fluid of fermented vegetables, herbs, fruits, cereals, beans, fungi, and seaweeds. ^2^ Japanese black vinegar (Kurozu), Moromi vinegar, or apple cider vinegar. ^3^ A powdered form of Japanese green vegetable juice, often containing young barley leaves, kale, or Ashitaba leaves. ^4^ Pu’er tea, Oolong tea, Tochu tea, or burdock tea.

**Table 7:** Channels of acquisition of products.

	20–29	30–39	40–49	50–59	60–69
Males, cumulative number	166	307	270	219	244
Stores	57.2	58.6	46.7	37.9	28.3
Online shopping (domestic)	25.9	30.3	43.3	49.8	62.3
Online shopping (overseas)	7.2	4.2	5.6	5.5	4.5
Friends (domestic)	3.6	2.3	0.7	2.3	3.7
Friends (overseas)	4.2	1.6	1.1	0.9	0
Others	1.8	2.9	2.6	3.7	1.2
Females, cumulative number	299	334	278	296	116
Stores	53.2	47.0	36.3	28.4	31.0
Online shopping (domestic)	33.4	41.3	56.8	64.2	64.7
Online shopping (overseas)	2.0	0.9	1.1	3.0	0.0
Friends (domestic)	3.3	2.7	1.8	1.7	2.6
Friends (overseas)	0.3	0.3	0	0	0
Others	7.7	7.8	4.0	2.7	1.7

Note: Multiple answers, expressed as percentage. The participants who did not remember the name of the product and manufacturer were included; thus, the total cumulative number was 1206 in males and 1323 in females. Males reported regarding sexual enhancement products; females reported regarding weight loss products.

**Table 8:** Prevalence of adverse event experience.

	Sexual Enhancement (926 Males)		Weight Loss (987 Females)		*p*-Value
	*n*	%	*n*	%	
Experienced adverse events; Yes	163	17.6	121	12.3	0.001
Symptoms ^1^					
Nausea or heartburn	31	19.0	31	25.6	0.183
Gastrointestinal symptoms ^2^	44	27.0	65	53.7	<0.001
Headaches	35	21.5	12	9.9	0.010
Cutaneous symptom	23	14.1	14	11.6	0.529
Warmth or redness in the face, neck, or chest	59	36.2	4	3.3	<0.001
Dizziness	15	9.2	5	4.1	0.099
Fatigue	16	9.8	9	7.4	0.484
Palpitation or shortness of breath	16	9.8	5	4.1	0.070
Others	7	4.3	5	4.1	0.946

Note: The differences among gender groups were examined by Chi-square test (*p* < 0.05). ^1^ multiple answers. ^2^ Diarrhea, constipation, or stomach pains.

**Table 9:** Attitudes toward consulting and reporting about adverse events related to sexual enhancement (males)/weight loss (females) supplements.

	Had Experienced Adverse Events		*p*-Value	Never Experienced Adverse Events ^1^		*p*-Value
	Sexual Enhancement (163 Males)	Weight Loss (121 Females)		Sexual Enhancement (763 Males)	Weight Loss (866 Females)	
Not consult with anybody	58.3	65.3	0.231	45.0	25.3	<0.001
It is too embarrassing ^2^	44.2	12.7	<0.001	38.5	10.0	<0.001
It is too much hassle ^2^	36.8	41.8	0.507	52.8	59.8	0.101
The symptom is temporal ^2^	51.6	53.2	0.835	14.3	31.5	<0.001
I can endure the cost of the effect ^2^	11.6	20.3	0.116	9.9	10.0	0.959
Others ^2^	2.1	10.1	0.024	5.5	9.1	0.102
Ask or complain to the manufacturer	8.0	5.8	0.476	20.1	35.2	<0.001
Ask or complain to the retail store	12.9	9.1	0.318	11.3	16.4	0.003
Consult with a partner	9.2			6.9		
Consult with family members, friends, relatives or colleagues	12.9	10.7	0.583	5.5	21.9	<0.001
Report the incident to governmental agencies ^3^	9.8	3.3	0.034	2.1	1.3	0.192
Report the incident to a public health center	6.1	5.8	0.902	3.9	7.2	0.005
Consult with a doctor (clinic)	1.2	3.3	0.228	24.8	30.7	0.008
Write on a message board or review site	4.3	3.3	0.669	4.7	7.0	0.048
Write on social media	2.5	1.7	0.643	2.2	3.5	0.137
Others	0.6	1.7	0.397	0.9	0.7	0.611

Note: Multiple answers, expressed as percentage. The differences among gender groups were examined by Chi-square test (*p* < 0.05). ^1^ Asked on the assumption that adverse events like palpitation or shortness of breath occurred. ^2^ Exclusively asked to those who answered “not consult with anybody”; the number of each group was as follows: Had experienced adverse events in males, 89; in females, 72; never experienced adverse events in males, 343; in females, 219. ^3^ Governmental agencies include the Ministry of Health, Labour and Welfare, the Consumer Affairs Agency, the Government of Japan, the National Consumer Affairs Center of Japan, and other consumer centers.

**Table 10:** The persons who users did not want to know about their sexual enhancement (males)/weight loss (females) supplement use.

	Had Experienced Adverse Events		*p*-Value	Never Experienced Adverse Events		*p*-Value
	Sexual Enhancement (163 Males)	Weight Loss (121 Females)		Sexual Enhancement (763 Males)	Weight Loss (866 Females)	
Nobody	21.5	52.1	<0.001	49.7	66.1	<0.001
Partner	37.4			25.6		
Family members or relatives	45.4	19.0	<0.001	32.8	13.3	<0.001
Friends	22.1	27.3	0.314	22.5	23.1	0.791
Colleagues	23.9	18.2	0.244	19.7	15.9	0.049
Doctors	9.8	5.0	0.130	4.3	2.9	0.118
Someone at the consultation services	5.5	0.0	0.009	4.5	0.8	<0.001
Others	0.6	1.7	0.397	0.5	1.7	0.024

Note: Multiple answers, expressed as percentage. The differences among gender groups were examined by Chi-square test (*p* < 0.05).
